# Homozygous *mdm2* SNP309 cancer cells with compromised transcriptional elongation at p53 target genes are sensitive to induction of p53-independent cell death

**DOI:** 10.18632/oncotarget.5312

**Published:** 2015-09-19

**Authors:** Melissa Rosso, Alla Polotskaia, Jill Bargonetti

**Affiliations:** ^1^ The Department of Biological Sciences Hunter College at The Belfer Research Building and The Graduate Center Biology PhD Program, CUNY, New York, NY 10021, USA

**Keywords:** MDM2, p53, chromatin, transcription elongation, 8-amino-adenosine

## Abstract

A single nucleotide polymorphism (T to G) in the *mdm2* P2 promoter, *mdm2* SNP309, leads to MDM2 overexpression promoting chemotherapy resistant cancers. Two *mdm2* G/G SNP309 cancer cell lines, MANCA and A875, have compromised wild-type p53 that co-localizes with MDM2 on chromatin. We hypothesized that MDM2 in these cells inhibited transcription initiation at the p53 target genes *p21* and *puma*. Surprisingly, following etoposide treatment transcription initiation occurred at the compromised target genes in MANCA and A875 cells similar to the T/T ML-1 cell line. In all cell lines tested there was equally robust recruitment of total and initiated RNA polymerase II (Pol II). We found that knockdown of MDM2 in G/G cells moderately increased expression of subsets of p53 target genes without increasing p53 stability. Importantly, etoposide and actinomycin D treatments increased histone H3K36 trimethylation in T/T, but not G/G cells, suggesting a G/G correlated inhibition of transcription elongation. We therefore tested a chemotherapeutic agent (8-amino-adenosine) that induces p53-independent cell death for higher clinically relevant cytotoxicity. We demonstrated that T/T and G/G *mdm2* SNP309 cells were equally sensitive to 8-amino-adenosine induced cell death. In conclusion for cancer cells overexpressing MDM2, targeting MDM2 may be less effective than inducing p53-independent cell death.

## INTRODUCTION

Chemotherapeutic agents that damage DNA activate the p53 pathway and can initiate cancer cell death [1, 2]. However, chemoresistant cancers often have sustained changes that block activation of the p53 pathway either by missense mutations in p53 or by overexpression of MDM2 [3]. The wild-type p53 protein is a tumor suppressor referred to as the “guardian of the genome” [4]. Under cellular stress conditions, such as DNA damage, wild-type p53 becomes activated and stabilized [5, 6]. The p53 protein functions as a transcription factor to induce transcriptional targets involved in activating cell cycle arrest, apoptosis, DNA repair and senescence (see reviews [6, 7]). Multiple proteins are involved in p53 regulation but one of the most critical negative regulators of p53 is the protein MDM2. The overexpression of MDM2 is found in multiple tumor types (see reviews [8, 9]). Therapies targeting the MDM2-p53 interaction are in development and considered to be promising (see reviews [10, 11]). However, it remains unclear if such therapies will be the most clinically relevant in all settings in which MDM2 is overexpressed.

MDM2 inhibits p53 activity by multiple mechanisms. MDM2 is an E3 ubiquitin ligase that targets p53 for proteasomal degradation [12] and can also inhibit p53 transactivation function on chromatin [13–16]. Increasing evidence demonstrates that MDM2 E3 ligase activity is controled by post-translational modifications on multiple domains of the polypeptide indicating that alterative MDM2 functions can coexist based on the kinase profile and splicing dynamics of the cell [17–20]. The *mdm2* gene itself is a transcriptional target of p53 and therefore p53 and MDM2 form a negative feedback loop [21–23]. The importance of the MDM2-p53 interaction is highlighted by the fact that the knockout of the *mdm2* gene in mice is embryonic lethal and is rescued by additonal knockout of *p53* [24]. MDM2 overexpression in cancers is associated with *mdm2* genomic amplification, increased transcription and enhanced translation [25–28]. One mechanism for increased transcription of *mdm2* is through a single nucleotide polymorphism at position 309 (*mdm2* SNP309) in which a thymine to guanine change increases recruitment of the transcription factor Sp1 to the genes P2 promoter [29]. Patients characterized as homozygous G/G *mdm2* SNP309 often have accelerated tumor formation, earlier age of cancer onset and increased incidence of multiple types of cancers [29, 30].

Human cancer cell lines that are G/G *mdm2* SNP309 are resistant to standard chemotherapeutic DNA damaging agents and have compromised p53 transcriptional activity after DNA damage treatment [14, 31]. Two human G/G SNP309 cancer cell lines, MANCA and A875, have stable wild-type p53 that is compromised for activation of multiple p53 target genes and forms MDM2-p53 chromatin complexes at p53 response elements [14]. MDM2 inhibits p53 transcriptional activity through dual mechanisms by binding to the p53 transactivation domain and TFIIE to inhibit the pre-initiation complex [13, 32]. However, recent evidence indicates that across the human genome silenced genes contain RNA polymerase II in functional pre-initiation complexes poised to begin transcription [33]. One p53 target gene, *p21*, can have blocked transcriptional elongation during an S-phase checkpoint [34–36], which may allow for rapid re-activation of the p53 pathway. In addition, the expression of MDM2 in SNP309 cancer cells also has p53-independent oncogenic activities [37, 38]. The list of p53-independent functions for MDM2 is increasing, with recent reports indicating that MDM2 has p53-independent transcription regulatory functions in Akt signaling [39]. This growing list suggests that overexpressed MDM2 might function to block activation of p53-mediated transcription in ways yet to be determined that do not require inhibition of transcription initiation. Alternatively, the overexpression of MDM2 from G/G SNP309 may be linked to other genetic or epigenetic changes that have not yet been identified.

In this study we explored the role of G/G SNP309 MDM2 overexpression on wild-type p53 transcriptional activity at *p21* and *puma* target genes. We tested if stable knockdown of MDM2 in G/G SNP309 cancer cells could reactivate wild-type p53. We found that MDM2 knockdown had a moderate activation effect on specific p53 target genes, including *puma*, in the absence of DNA damage treatment but did not induce *p21*. Importantly, in the presence of DNA damage, MANCA and A875 cells had functional transcription initiation at the p53 target genes *p21* and *puma* but had compromised transcriptional elongation. We found it difficult to reactivate the initiated wild-type p53 causing us to ask the clinically relevant question of what is the best way to reduce the viability of G/G SNP309 cancer cells?

Inducers of p53-independent cell death can work on multiple cancer types with or without p53 mutations, therefore activating p53-independent cell death is potentially more clinically relevant than inhibiting the MDM2 pathway [40–42]. Many cancers overexpress MDM2, but also express mutant p53 that is unable to activate the transcription of death inducing target genes [38, 43, 44]. For example, many triple negative breast cancers express high MDM2 as well as mutant p53 [45]. We have recently found that triple negative breast cancers with mutant p53 are killed effectively by the p53-independent death inducer called 8-amino-adenosine (8AA) [41]. The cytotoxic effects of 8AA occur by inhibiting RNA metabolism, reducing the pools of ATP, and blocking Akt/mTOR signaling [46]. Actinomycin D which represses RNA Pol1 activity and reduces rRNA transcription, at extremely low doses can directly inhibit MDM2 by releasing ribosomal proteins that inhibit MDM2 thereby activating the p53 pathway [47]. To date no study has been undertaken to compare how cells with overexpressed MDM2 through *mdm2* SNP309 are killed by activation of p53-dependent versus p53-independent pathways. In theory, G/G *mdm2* SNP309 cells that express wild-type p53 should be killed by blocking MDM2. However, in practice cancers are polymorphic and G/G *mdm2* SNP309 cancers may select for additional pathways to inactive wild-type p53. Recent evidence implicates the activation of MDMX as an alternative mechanism for cancers to inactive the wild-type p53 pathway [48, 49]. In MDM2 overexpressing cancers, it may be more clinically relevant to initiate p53-independent cell death pathways because it is unclear how high-level wild-type p53 mediated transcriptional activation is blocked.

When cancers are resistant to standard chemotherapy it is important to consider alternative targeting options. Cancers with high MDM2 are sometimes, but not always, sensitive to small molecule chemotherapeutics disrupting the p53-MDM2 interaction (see reviews [11, 40]). Non-genotoxic small molecule inhibitors targeting this interaction such as Nutlin-3 are reported to have some efficacy in cancers with MDM2 overexpression [40, 50]. Interestingly, herein we found that knockdown of MDM2 was not able to induce death in G/G SNP309 cancer cells, suggesting the need to determine other targeted treatments for such MDM2 overexpressing cancers. Specific activation of wild-type p53 by low dose actinomycin D treament has been suggested as a clinically relevant treatment option for cancers with high MDM2 [51]. However, we found that while actinomycin D treatment increased p53 levels in G/G SNP309 cancer cells, this treatment did not substantially decrease cell viability. Interestingly, we observed that the nucleoside analogue 8AA, which activates p53–independent cell death pathways [41], was more cytotoxic to G/G SNP309 cancer cells than etoposide or actinomycin D suggesting it is a viable option for cancers with dysfunctional p53. Cancers with wild-type p53 and high MDM2 are potentially well suited as candidates for treatments targeted at p53-independent death pathways. Synthetic lethal p53-independent cell death pathways are emerging as important targets for multiple cancer types [52]. In the clinical setting, activation of p53-independent cell death pathways may be the best target for *mdm2* G/G SNP309 cancers.

## RESULTS

### Cancer cell lines with the G/G SNP309 genotype have compromised transcription of p53 target genes

**Figure 1:**
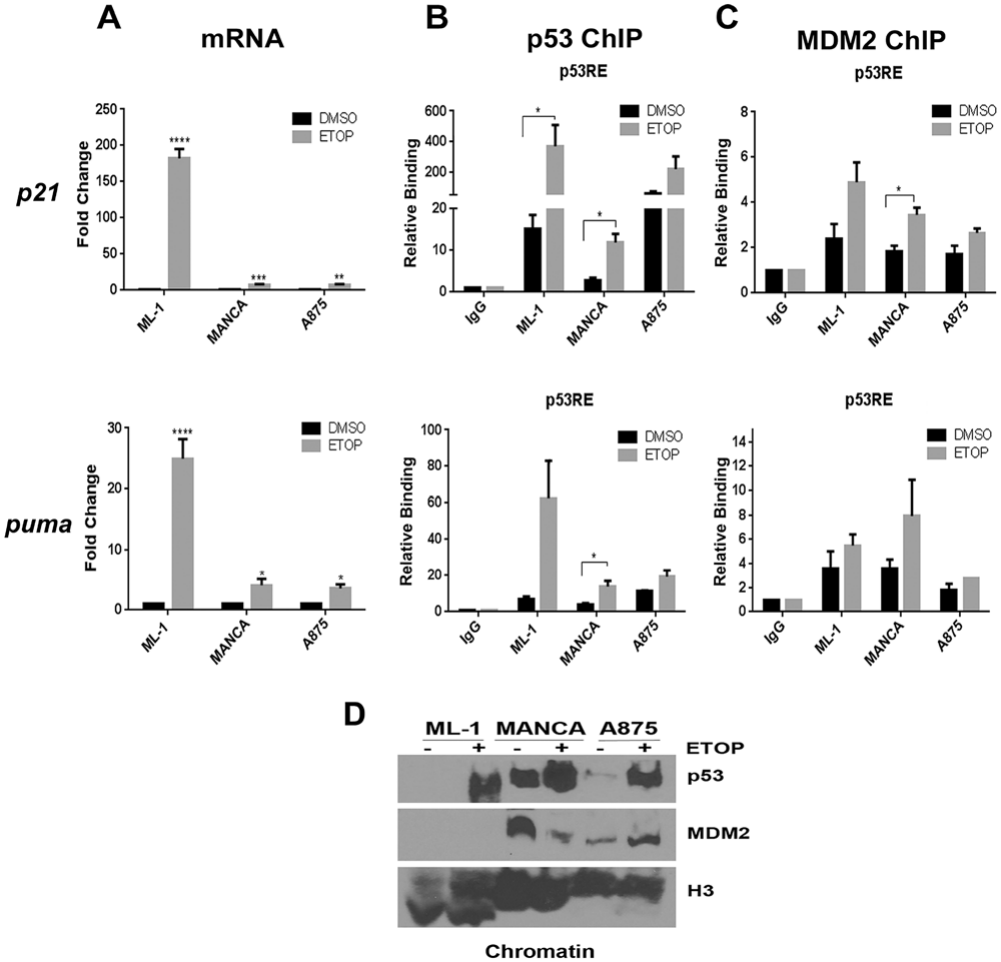
Cancer cells with G/G *mdm2* SNP309 have compromised transcriptional activation of p53 target genes after DNA damage ML-1, MANCA and A875 cells were treated with 8 μM etoposide (ETOP) for 6 hours. **A.** *p21* and *puma* transcript was measured using quantitative RT-PCR. Samples were first normalized to DMSO for target gene expression and then to total *gapdh* mRNA. Results represent an average of three to five independent experiments given with standard error bars. Student *t* test analysis of cells treated DMSO vs ETOP for ML-1[*p21* and *puma p* < 0.0001], MANCA [*p21 p* = 0.0002, *puma p* = 0.019] and A875 [p21 *p* = 0.004, *puma p* = 0.021]. One way ANOVA analysis compared ML-1, MANCA and A875 cells treated with ETOP for *p21* and *puma* [*p* < 0.0001]. Student *t*-test analysis also used for comparison between cell lines treated with ETOP for *p21* [ML-1 vs. MANCA *p* = 0.00015, ML-1 vs. A875 *p* = 0.00014] and *puma* [ML-1 vs. MANCA *p* = 0.0019, ML-1 vs. A875 *p* = 0.002]. **B.** Chromatin immunoprecipitations were performed for p53 protein and analyzed by quantitative PCR using primers for the *p21* gene 5′ p53RE and *puma* gene p53RE. Student *t*-test analysis compared cells treated with DMSO vs. ETOP for ML-1 [*p21 p* = 0.045, *puma p =* 0.053], MANCA [*p21 p* = 0.005, *puma p* = 0.023] and A875 [*p21 p* = 0.198, *puma p =* 0.15]. **C.** Chromatin immunoprecipitations were performed for MDM2 protein and analyzed by qPCR using primers as described in B. Student *t*-test analysis compared cells treated with DMSO vs. ETOP for ML-1 [*p21 p* = 0.052, *puma p* = 0.30], MANCA [*p21 p* = 0.004, *puma p* = 0.198] and A875 [*p21 p* = 0.166, *puma p* = 0.184]. ML-1 and MANCA results represent four to six independent experiments. A875 results represent two independent experiments. All chromatin immuno-precipitations are normalized to IgG and input values. **D.** 50 μg of chromatin extract was subjected to SDS-PAGE and western blot analysis. A representative image is shown.* represents a *p* value ≤ 0.05, ** represents *p* value ≤ 0.01, *** represents a *p* value ≤ 0.001, **** represents *p* value ≤ 0.0001.

We compared p53 signaling in MDM2 SNP309 T/T and G/G genotype cells by using the previously documented model of comparison of ML-1, MANCA and A875 human cancer cell lines [14, 29]. ML-1 cells, a myeloid leukemia, have functional wild-type p53 and basal MDM2 protein expression with a T/T *mdm2* SNP309 genotype [29]. MANCA and A875 cell lines, a Burkitt's lymphoma and melanoma (respectively), have compromised wild-type p53 and overexpression of MDM2 due to the G/G *mdm2* SNP309 genotype [29]. MANCA and A875 cancer cells, compared to ML-1 cells, have compromised p53 transcriptional activity [14]. Using the established DNA damage conditions, we compared MANCA and A875 cells to ML-1 cells for activation of the previously investigated *p21* gene and for the first time the apoptotic target *puma*. As expected, DNA damage induced by 8 μM etoposide treatment in ML-1 cells stimulated robust *p21* and *puma* transcription. However, activation of *p21* was significantly less abundant in MANCA (*p* = 0.00015) and A875 (*p* = 0.00015) cells than observed in ML-1 cells. This was also observed for activation of *puma* in MANCA (*p* = 0.00018) and A875 (*p* = 0.002) cells (Figure 1A). These data supported our previous findings that cells with *mdm2* G/G SNP309 have compromised wild-type p53 activity.

DNA damage induced recruitment of p53 and MDM2 to the promoter regions of the two target genes in the three cell lines (Figure 1). The highest level of p53 recruitment was found in ML-1 cells at the *p21* promoter region (Figure 1B). Etoposide treatment increased p53 recruitment in MANCA and A875 cells at the *p21* and *puma* genes p53 responsive elements (p53REs) (Figure 1B). MANCA cells displayed significant increases in p53 recruitment after DNA damage for *p21* (*p* = 0.005) and *puma* (*p* = 0.023) genes. We observed some MDM2 recruitment in ML-1 cells at *p21* and *puma* genes that associated with the large increase in p53 recruitment (Figure 1C). Importantly, at the *p21* gene there was a significant increase in MDM2 recruitment in MANCA cells after DNA damage corresponding to the increased p53 (Figure 1C). This trend was also seen at the *puma* gene (Figure 1C). A875 cells also displayed a trend of increased MDM2 recruitment that corresponded to the p53 recruitment for both genes (Figure 1C). To compare overall levels of p53 and MDM2 on chromatin, we performed a chromatin fractionation and western blot analysis. We observed that in ML-1, MANCA and A875 cells etoposide treatment caused increased p53 protein levels on the chromatin (Figure 1D). Interestingly, MANCA cells had the highest basal levels of p53 on the chromatin. Additionally, MANCA and A875 cells had higher basal levels of MDM2 protein on the chromatin compared to ML-1 cells (Figure 1D). These data were in keeping with our previous findings [14] and suggested that stable knockdown of MDM2 in G/G SNP309 cells would result in activation of p53 target genes.

### MDM2 knockdown in G/G SNP309 cancer cells moderately increases p53 transcriptional activity without affecting p53 degradation

**Figure 2:**
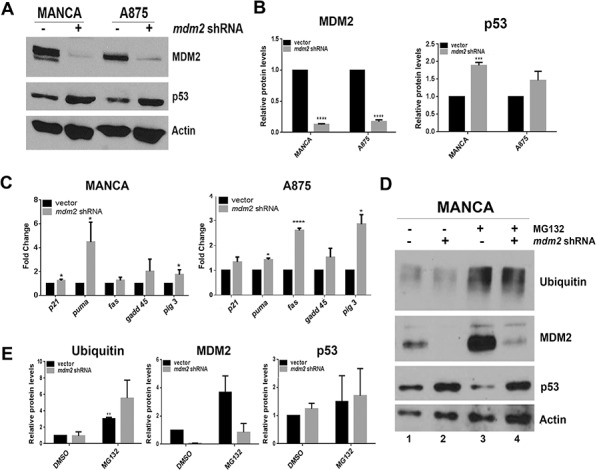
MDM2 knockdown in G/G SNP309 cancer cells moderately increases transcription of p53 target genes without increasing p53 degradation **A.** 50 μg of whole cell extract protein was run on 10% SDS-PAGE and Western blot analysis. A representative image is shown. **B.** Image J analysis was performed for MDM2 and p53 protein levels normalized to Actin. Graphs represent three independent experiments given with standard error bars. Student *t*-test analysis compared vector control and *mdm2* shRNA cell lines for MDM2 [MANCA and A875 *p* < 0.0001] and p53 [MANCA *p =* 0.0005, A875 *p* = 0.21] **C.** Quantitative RT-PCR was performed for five p53 target genes in both MANCA and A875 cell lines. Results were normalized to *gapdh* and represent three independent experiments given with standard error bars. Student *t* test analysis compared vector and *mdm2* shRNA values for MANCA [*p21 p* = 0.017, *puma p* = 0.022, *fas p* = 0.18, *gadd*45 *p =* 0.15, *pig* 3 *p =* 0.037] and A875 [*p21 p =* 0.17, *puma p =* 0.002, *fas p* < 0.0001, *gadd*45 *p =* 0.22, *pig* 3 *p* = 0.008] **D.** Cells were treated with 10 μM MG132 for 6 hours. Whole cell extracts were made and subjected to SDS-PAGE and western blot analysis. A representative image is shown. **E** Imaje J analysis represents three independent experiments for ubiquitin, MDM2 and p53 and normalized to Actin. Student *t*-test analysis compared MANCA vector DMSO to MG132 treatment for ubiquitin [*p =* 0.004], MDM2 [*p* = 0.15] and p53 [*p =* 0.63]. * represents a *p* value ≤ 0.05, ** represents *p* value ≤ 0.01, *** represents a *p* value ≤ 0.001, **** represents *p* value ≤ 0.0001.

In order to directly test the influence of MDM2 on p53 protein levels and activity in G/G SNP309 cancer cells we constructed MANCA and A875 stable mir30-based *mdm2* shRNA knockdown cell lines (Figure 2A). We observed a significant knockdown of 80 to 90% of MDM2 protein (Figures 2A and 2B). In MANCA cells, this resulted in a moderate and significant increase in p53 protein levels (Figures 2A and 2B). In A875 cells, there was a trend of increased p53 protein levels (Figures 2A and 2B). Surprisingly, the reduction of MDM2 did not translate into a global increase in transcription of p53 target genes as indicated by quantitative RT-PCR analysis of transcripts of five common p53 targets (Figure 2C). Interestingly, there was a cell type specific increase in subsets of transcripts. Stable MDM2 knockdown in MANCA cells increased *puma* by 4.5 fold and *pig 3* by 1.7 fold (Figure 2C). In A875 cells, the knockdown of MDM2 increased *fas* by 2.6 fold, *pig 3* by 2.9 fold and *puma* by 1.4 fold (Figure 2C). When MANCA and A875 cells with MDM2 knockdown were treated with etoposide for six hours, there was no additive effect on activation of the p53 target genes (data not shown). These data indicate that subsets of p53 target genes are sensitive to MDM2 knockdown in a cell-type specific manner and that inhibition of the *p21* target gene's transcription is strongly programmed.

MDM2 is known to be involved in mediating p53 degradation through the ubiquitin proteasome pathway [12]. However, in G/G SNP309 cancer cells degradation of p53 is not robust. We reasoned that if MDM2 knockdown were blocking ubiquitin-mediated proteasome degradation then addition of the proteasome inhibitor MG132 would increase p53 levels. In order to directly test this, we used MANCA cells because with MDM2 knockdown they had a significant increase in p53 protein levels. We treated MANCA cells with and without MDM2 shRNA knockdown with 10 μM MG132. The addition of MG132 increased the overall pool of ubiquitinated protein (Figures 2D and 2E) and increased MDM2 protein levels confirming that MDM2 is a target of the ubiquitin-proteosome pathway [53] (Figure 2D compare lanes 1 and 2 to lanes 3 and 4). MDM2 can function as an E3-ubiquitin ligase for itself [54] and our data suggest this may be the case in MANCA cells. Interestingly, MG132 treatment did not significantly increase p53 protein levels (Figure 2D compare lanes 1 and 3 and see Figure 2E). Moreover the combined MDM2 knockdown and MG132 treatment did not result in increased p53 protein levels compared to MDM2 knockdown alone (Figure 2D, compare lanes 2 and 4 and see Figure 2E). This demonstrated that in MANCA cells MDM2 is not targeting p53 for ubiquitin-mediated degradation. The increased p53 that occurred following MDM2 knockdown must result from an alternative mechanism.

### G/G SNP309 cancer cells have functional transcription initiation of p53 target genes after DNA damage

**Figure 3:**
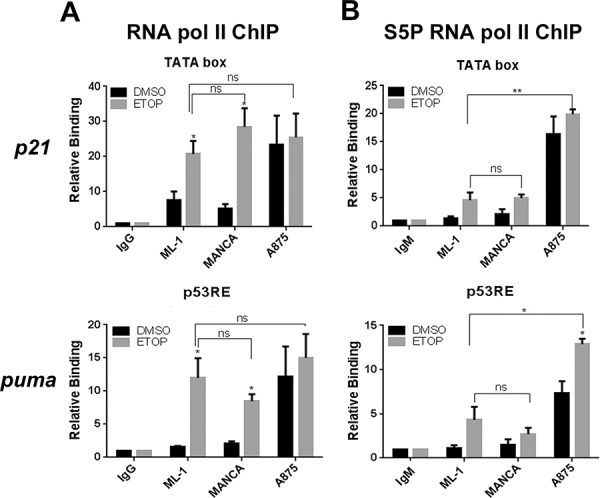
Etoposide treatment in G/G SNP309 cells results in functional transcription initiation ML-1, MANCA and A875 cells were treated with 8 μM etoposide (ETOP) for 6 hours. **A.** Total RNA polymerase II chromatin immunoprecipitations were carried out and analyzed using quantitative PCR (qPCR) primers specific to the *p21* transcription start site (TATA box) and the *puma* p53 responsive elements located near the p53 responsive transcription start site near exon 1a. All results normalized to IgG background and inputs from each cell line. Results represent six independent experiments for ML-1 and MANCA and four independent experiments for A875 cells. The results are presented with standard error bars. One-way ANOVA analysis compared ML-1, MANCA and A875 cells treated with ETOP for *p21* [*p =* 0.59] and *puma* [*p =* 0.55]. Student *t*-test used for comparison between cell lines treated with ETOP for *p21* [ML-1 vs. MANCA *p =* 0.26, ML-1 vs.A875 *p =* 0.41] and *puma* [ML-1 vs. MANCA *p* = 0.31, ML-1 vs. A875 *p =* 0.54]. The “ns” stands for non-significant. **B.** Phospho-Serine 5 (S5P) RNA polymerase II chromatin immunoprecipitations were carried out and analyzed with qPCR primers used in B. All results normalized to IgM and inputs. Results represent three independent experiments for ML-1 and MANCA cells and four independent experiments for A875 cells. The results are presented with standard error bars. One way ANOVA analysis compared ML-1, MANCA and A875 cells treated with ETOP for *p21* [*p* < 0.0001] and *puma* [*p =* 0.00019]. Student *t*-test analysis used for comparison between cell lines treated with ETOP for *p21* [ML-1 vs. MANCA *p =* 0.81, ML-1 vs. A875 *p =* 0.0015] and *puma* [ML-1 vs. MANCA *p =* 0.40, ML-1 vs. A875 *p =* 0.017]. All chromatin immuno-precipitations were normalized to IgM and input values. * represents a *p* value ≤ 0.05, ** represents *p* value ≤ 0.01, *** represents a *p* value ≤ 0.001, **** represents *p* value ≤ 0.0001.

In G/G SNP309 cancer cells, chemoresistance to DNA damage and attenuated p53 function are linked [29]. This attenuated wild-type p53 function is likely due to compromised p53 transcriptional activity [14]. Therefore, we asked if in G/G SNP309 cells after DNA damage there was compromised transcription initiation. In order to test this we compared the recruitment of RNA Pol II in ML-1 cells to the recruitment of Pol II seen in MANCA and A875 cells before and after etoposide treatment. In ML-1 cells, at the transcription start sites of both the *p21* and *puma* genes there was an increase in total RNA Pol II recruitment (Figure 3A). Importantly, etoposide treatment also increased RNA Pol II recruitment in MANCA cells (Figure 3A). Interestingly, the basal RNA Pol II recruitment in A875 cells was high before DNA damage treatment and etoposide treatment did not cause a substantial increase (Figure 3A). We determined with statistical analysis that after etoposide treatment all cell lines tested had comparable RNA Pol II recruitment at *p21* and *puma* transcription start sites (Figure 3A). These data indicated that MANCA and A875 cells had functional transcription initiation for *p21* and *puma* target genes.

To further confirm that etoposide treatment in SNP309 G/G cells resulted in transcription initiation we analyzed the recruitment of an initiated form of RNA Pol II. The C-terminal (CTD) tail of RNA Pol II consists of 52 heptapeptide (YSPTSPS) repeats, which when phosphorylated at position 5 (serine 5) represent an initiated form of Pol II (see reviews [55, 56]). We observed that following etoposide treatment there was a comparable recruitment of phosphorylated serine 5 RNA Pol II in ML-1 and MANCA cells at both *p21* and *puma* genes (Figure 3B). As seen with total RNA Pol II recruitment, there was high basal recruitment of the phosphorylated serine 5 RNA Pol II in A875 cells for *p21* and *puma* genes (Figure 3B). Additionally, there was a significant increase in the initiated form of RNA Pol II after DNA damage in A875 cells at the *puma* gene (Figure 3B). These data provided evidence that despite compromised transactivation of *p21* and *puma* in G/G *mdm2* SNP309 cells, both genes demonstrated functional transcription initiation.

### Decreased H3K36 trimethylation in G/G SNP309 cancer cells demonstrates less active transcription elongation at p53 target genes after DNA damage

**Figure 4:**
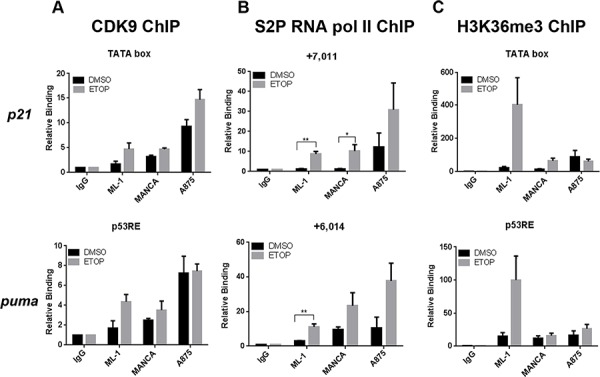
Reduced Histone H3K36me3 indicates less active transcription elongation near p53 target gene transcription start sites ML-1, MANCA and A875 cells treated with DMSO or 8 μM etoposide (ETOP) for 6 hours. **A.** CDK9 chromatin immunoprecipitations were carried out and analyzed using qPCR primers to transcription start sites for *p21* and *puma* genes. Results represent four independent experiments for ML-1 cells and three independent experiments for MANCA and A875 cells. The results are presented with standard error bars. One way ANOVA analysis comparing ML-1, MANCA and A875 cells treated with ETOP for *p21* [*p =* 0.02] and *puma* [*p =* 0.027]. Student *t*-test analysis used to compare cells treated with ETOP for *p21* [ML-1 vs. MANCA *p =* 0.96, ML-1 vs. A875 *p =* 0.02] and *puma* [ML-1 vs. MANCA *p =* 0.50, ML-1 vs. A875 *p =* 0.030]. **B.** Phospho-Serine 2 RNA pol II chromatin immunoprecipitations were carried out and analyzed using qPCR primers to *p21* +7011 and *puma* +6014 regions of the genes. Student *t* test analysis compared cells treated DMSO vs ETOP for ML-1 [*p21 p =* 0.005, *puma p =* 0.009], MANCA [*p21 p =* 0.047, *puma p =* 0.15] and A875 [*p21 p =* 0.29, *puma p =* 0.085]. Results represent three independent experiments with standard error bars. **C.** H3K36me3 chromatin immunoprecipitations were carried out and analyzed by qPCR primers for *p21* and *puma* genes transcription start sites. One way ANOVA analysis for ML-1, MANCA and A875 cells treated with ETOP for *p21* [*p =* 0.052] and *puma* [*p =* 0.04]. Student *t* test analysis used for comparison between cell lines treated with ETOP for *p21* [ML-1 vs. MANCA *p =* 0.133, ML-1 vs. A875 *p =* 0.130] and *puma* [ML-1 vs. MANCA *p =* 0.102, ML-1 vs. A875 *p =* 0.133]. The difference in the means after ETOP treatment for ML-1 and MANCA is (338 with 95% Confidence Interval (CI) [39–638]) and between ML-1 and A875 is (342 with 95% CI [43–642]). Results represent four independent experiments with standard error bars. All chromatin immunoprecipitations samples are normalized to IgG and input values. * represents a *p* value ≤ 0.05, ** represents *p* value ≤ 0.01, *** represents a *p* value ≤ 0.001, **** represents *p* value ≤ 0.0001.

The recruitment of Ser5 CTD Pol II indicated that promoter clearance had occurred and that the block might be at the level of transcriptional elongation. We therefore asked if the active subunit of the positive transcription elongation factor P-TEFb, CDK9, was recruited to *p21* and *puma* genes. CDK9 phosphorylates the CTD of RNA Pol II primarily at serine on position two (serine 2) of its heptapeptide sequence, thereby transitioning the transcription machinery into productive transcriptional elongation [55]. Using chromatin immunoprecipitation analysis, we observed that ML-1, MANCA and A875 cell lines had comparable CDK9 recruitment at *p21* and *puma* transcription start sites (TSS) after DNA damage (Figure 4A). This indicated that CDK9 was available to promote transcription elongation. We also examined the recruitment of the elongating phospho-serine 2 CTD RNA Pol II. Since phosphorylation of serine 2 on RNA Pol II CTD increases toward the 3′ end of genes [57], we looked at regions of *p21* (+7011) and *puma* (+6014) genes located near exon 3. In ML-1 cells, etoposide treatment caused a significant increase in phospho-serine 2 RNA Pol II recruitment (Figure 4B). In etoposide treated MANCA and A875 cells, downstream of *p21* and *puma* TSS, we also detected an increased recruitment of phospho-serine 2 RNA Pol II (Figure 4B). These cell lines displayed trends of increased elongating Pol II recruitment after DNA damage and a significant increase on the *p21* gene in MANCA cells (Figure 4B). However, the level of full length transcript from both the *p21* and *puma* genes was compromised (Figure 1A).

To further analyze the elongation signature at the chromatin level, we compared histone H3 Lysine 36 trimethylation (H3K36me3), a histone post-translational modification mark associated with active transcriptional elongation [58]. H3K36me3 has also been linked to communication between chromatin and pre-mRNA processing which can influence the rate of transcription [59–61]. We expected that following DNA damage in ML-1 cells we would detect an increase in H3K36me3 that would correlate with the activated transcription of p53 target genes. We observed that at the TSS for *p21* and *puma* genes there was an increase in H3K36me3 in ML-1 cells after DNA damage treatment (Figure 4C). In comparison to ML-1 cells after etoposide treatment, MANCA and A875 cells showed a strong trend of less H3K36me3 at *p21* and *puma* genes (Figure 4C). A large difference in the means was observed when comparing H3K36me3 between ML-1 and MANCA and between ML-1 and A875 after DNA damage. Together the data show that MANCA and A875 produce less full-length *p21* and *puma* mRNA and this correlates with a decrease in H3K36me3. This indicated that although transcription initiation was intact and some elongation marks were maintained the rate of elongation was slower and might have been influenced by an H3K36me3 signature.

**Figure 5:**
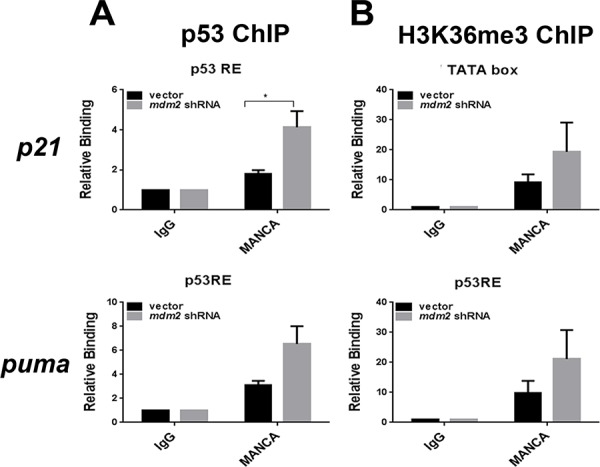
MDM2 knockdown in MANCA cells increases p53 recruitment without altering H3K36me3 at p53 target gene transcription start sites **A.** Chromatin immunoprecipitations were performed for p53 protein and analyzed by quantitative PCR using primers for the *p21* gene 5′ p53 responsive element and *puma* gene p53 responsive elements. Student *t*-test analysis performed comparing vector vs. *mdm2* shRNA for *p21* [*p* = 0.029] and *puma* [*p* = 0.085]. Results represent four independent experiments given with standard error bars. **B.** H3K36me3 chromatin immunoprecipitations were carried out and analyzed by qPCR primers for *p21* and *puma* gene transcription start sites. Student *t* test analysis compared vector vs. *mdm2* shRNA for *p21* [*p* = 0.34] and *puma* [*p* = 0.314]. Results represent five independent experiments given with standard error bars. All chromatin immunoprecipitations are normalized to IgG and input values. * represents a *p* value ≤ 0.05.

To further explore the nature of the H3K36me3 signature we addressed the influence of stable MDM2 knockdown on the chromatin recruitment of p53 and H3K36me3 on the *p21* and *puma* genes. In MANCA cells, we observed a significant increase in p53 recruitment at the *p21* (*p* = 0.029) gene and a trend of increased p53 recruitment at the *puma* (*p* = 0.085) gene (Figure 5A). The correlation between G/G SNP309 and reduced H3K36me3 led us to ask if MDM2 knockdown could reverse the epigenetic phenotype. Upon depletion of MDM2, we did not detect a significant increase in H3K36me3 (Figure 5B). This indicated that while high MDM2 levels were directly related to reduced p53 recruitment it might not be directly involved in the inhibition of H3K36me3 recruitment. Our work clearly demonstrated that stimulating p53 recruitment to chromatin by DNA damage treatment or targeted knockdown of MDM2 did not overcome the compromised increase of *p21* and *puma* transcripts. This suggested that restoring compromised wild-type p53 function by small molecule inhibitors of the p53-MDM2 interaction might be difficult in chemoresistant MDM2 overexpressing cancers.

### Alternative treatment options for G/G SNP309 chemoresistant cancer cells with compromised wild-type p53

Our attempts to reactivate the p53 pathway with MDM2 knockdown or etoposide-mediated DNA damage in G/G SNP309 cancer cells did not result in robust target gene transcription or induction of cell death. Reactivation of the wild-type p53 pathway using these methods is not likely to be the best option for treating such chemoresistant cancers. We therefore explored alternative pathways for inducing cell death in G/G SNP309 MDM2 overexpressing cells. Inhibitors of general transcription and RNA synthesis have been sought as alternative approaches for cancer treatment (see review [62]).

**Figure 6:**
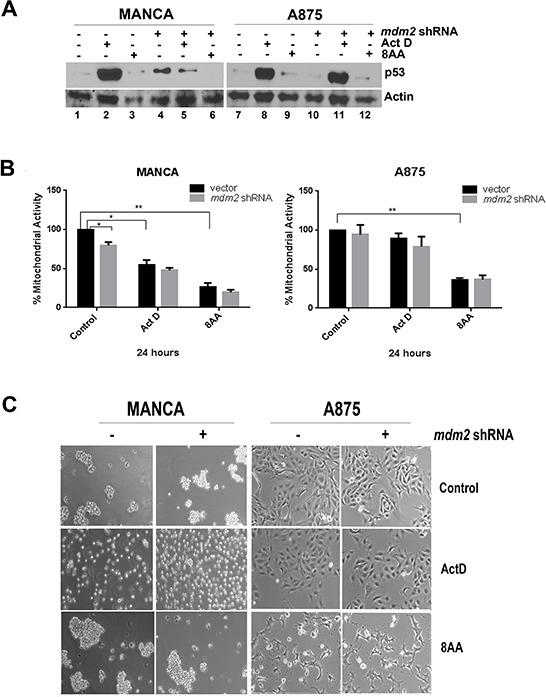
Chemoresistant G/G SNP309 cancer cells are sensitive to 8-amino-adenosine MANCA and A875 with constitutively expressed *mdm2* shRNA or vector control were treated with 5 nM Actinomycin D (Act D) or 15 μM 8-amino-adenosine (8AA) for 24 hours. **A.** 50 μg of whole cell extracts protein was subjected to SDS-PAGE and western blot analysis. A representative image is shown. **B.** The MTT assay was performed on the cells after treatment. Results represent three independent experiments with standard error bars. Student *t*-test analysis compared control to either drug treatment for MANCA vector [Act D *p =* 0.018, 8AA *p =* 0.005] and A875 vector [Act D *p* = 0.22, 8AA *p* = 0.008]. MANCA vector vs *mdm2* shRNA [*p =* 0.036]. **C.** After treatment, images of cells under 10X objective were taken. * represents a *p* value ≤ 0.05, *** represents *p* value ≤ 0.001, **** represents *p* value ≤ 0.0001.

Actinomycin D interferes with the elongation of RNA in actively transcribing genes [63]. At low doses, actinomycin D is reported to reactivate the p53 pathway via MDM2 binding to ribosomal proteins released from the nucleolus and preventing p53 degradation [51, 64]. We compared MANCA and A875 cells treated with 5nM actinomycin D for 24 hours to MANCA and A875 cells with MDM2 knockdown (Figure 6). Actinomycin D treated MANCA and A875 cells had a robust increase in p53 protein levels as compared to cells with no treatment (Figure 6A lanes 1 and 2, 7 and 8). Interestingly, MANCA cells with MDM2 knockdown had p53 protein at lower levels as compared to actinomycin D treatment alone (Figure 6A, compare lanes 2 and 5). In A875 cells treated with actinomycin D, the levels of p53 remained about the same with or without MDM2 knockdown (Figure 6A, compare lanes 8 and 11). Actinomycin D treatment failed to reduce the viability of A875 cells as assessed by the MTT assay; and the same assay showed a 50% decrease in MANCA cell mitochondrial activity (Figure 6B). In keeping with the MTT assay, microscopic examination demonstrated that while actinomycin D treated MANCA cells were unhealthy and stopped aggregating, the A875 cells remained healthy (Figure 6C). This provides further evidence that simply increasing the level of p53 is not sufficient to kill chemoresistant MDM2 overexpressing cancer cells.

The nucleoside analog 8-amino-adenosine (8AA), interferes with general transcription and RNA metabolism in a p53-independent manner [41, 65]. We asked if treating chemoresistant G/G SNP309 cancer cells with 8AA would reduce cell viability better than traditional p53 reactivation mechanisms. MANCA and A875 cells were treated with a concentration of 8AA previously reported to activate cell death in several metastatic breast cancer cells [41]. The p53 protein levels in the treated cells did not increase with 8AA treatment (Figure 6A lanes 1 and 3; 7 and 9). We observed that 8AA treated MANCA and A875 cell lines had a significant reduction in mitochondrial activity and looked sick when examined by light microscopy (Figures 6B and 6C). The knockdown of MDM2 protein, in addition to 8AA treatment, did not significantly alter cell viability. This finding suggested that inducing p53-independent cell death in MDM2 overexpressing cells might be the best strategy for G/G SNP309 cancers with compromised wild-type p53 function.

### Comparison of 8AA and low dose actinomycin D for activation of death in T/T and G/G *mdm2* SNP309 cancer cells

**Figure 7:**
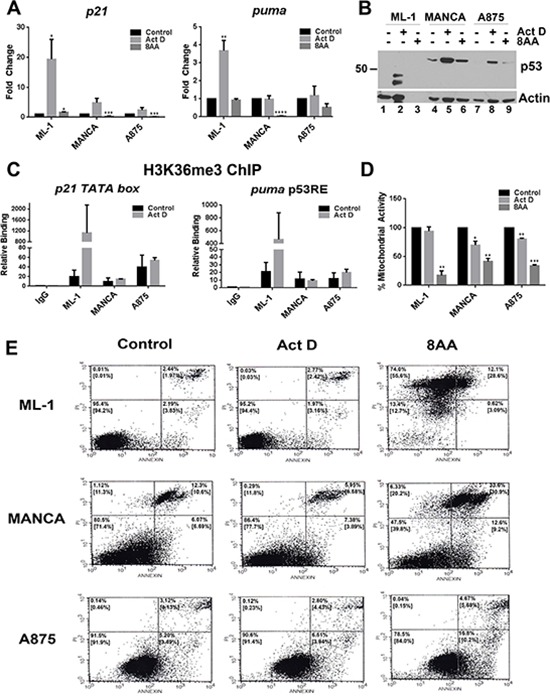
8-Amino-adenosine is more cytotoxic to T/T and G/G mdm2 SNP309 cancer cells than low dose actinomycin D treatment ML-1, MANCA and A875 cells were treated as in Figure 6 (A-D for 24 hours, E for 16 hours). **A.** Quantitative RT-PCR was performed for *p21* and *puma* genes. All results were normalized to *gapdh*. For Act D treatment, the results represent three independent experiments with standard error bars. Student *t*-test analysis was done to compare Act D treatment for *p21* [ML-1 *p* = 0.049, MANCA *p* = 0.055, A875 *p =* 0.18] and *puma* [ML-1 *p* = 0.009, MANCA *p* = 0.87, A875 *p* = 0.75]. For 8AA treatment, the results represent two independent experiments with standard error bars. Student *t*-test analysis done for comparison of 8AA treatment for *p21* [ML-1 *p* = 0.017, MANCA *p* = 0.001, A875 *p* = 0.0004] and *puma* [ML-1 *p* = 0.316, MANCA *p* < 0.0001., A875 *p* = 0.056]. **B.** 50 μg of whole cell extracts protein was subjected to SDS-PAGE and western blot analysis. A representative image is shown. **C.** H3K36me3 chromatin immunoprecipitations were carried out for Act D treated cells and analyzed using qPCR with primers to *p21* and *puma* transcription start sites. The results represent two independent experiments with standard error bars. Student *t*-test analysis was done to compare Act D treated cells for *p21* [ML-1 *p* = 0.391, MANCA *p* = 0.591, A875 *p* = 0.646] and *puma* [ML-1 *p* = 0.398, MANCA *p* = 0.854, A875 *p* = 0.465]. **D.** The MTT assay was performed on the cells after treatment. The results represent two independent experiments with standard error bars. Student *t*-test analysis done for comparison of Act D treatment for ML-1[*p* = 0.51], MANCA [*p* = 0.045] and A875 [*p* = 0.006]. Student *t*-test analysis also done for comparison of 8AA treatment for ML-1[*p* = 0.0089], MANCA [*p* = 0.0085] and A875 [*p* = 0.00045] cells. **E.** To assess for apoptosis, the cells were treated for 16 hours and stained with Annexin V and propidium iodide followed by analysis with flow cytometry. The lower left quadrant shows unstained cells, the lower right shows Annexin V-positive cells, the upper left quadrant shows propidium iodide-positive cells and the upper right quadrant shows Annexin V and propidium iodide-positive cells. The percentages in the upper left and right hand quadrant corners represent the values for the representative experiment shown. The numbers in the brackets represent the average of two independent experiments. * represents a *p* value ≤ 0.05,** represents *p* value ≤ 0.01, *** represents *p* value ≤ 0.001, **** represents *p* value ≤ 0.0001.

We compared T/T and G/G *mdm2* SNP309 cancer cells for their outcomes after treatment with low dose actinomycin D or 8AA in order to compare p53-dependent versus p53-independent cell death (Figure 7). We observed that low dose actinomycin D treatment activated transcription of the p53 target genes *p21* and *puma* in T/T ML-1 cells, but not in the G/G MANCA or A875 cells (Figure 7A). Furthermore, 8AA did not activate p53 target gene transcription and in some cases caused a decrease (Figure 7A). To determine if the increase in p53 transcriptional activity correlated with stabilized p53 protein, we performed western blot analysis on whole cell extracts for control and actinomycin D treated cells (Figure 7B). For actinomycin D treatment, no direct correlation was observed between p53 levels and activation of transcription, which recapitulated the data shown in Figure 1. Actinomycin D increased p53 protein levels in ML-1, MANCA, and A875 cells (Figure 7B, compare lane 1 to 2, lane 4 to 5, and lane 7 to 8 respectively). However transcription was only activated in ML-1 cells (Figure 7A). Therefore, compared to T/T ML-1 cells, G/G SNP309 MANCA and A875 cells displayed compromised transcription of p53 target genes. The molecular weight of the p53 bands in ML-1 extract were reproducibly lower than expected, while the p53 in MANCA and A875 cells was the predicted size (Figure 7B, lanes 2, 5 and 8). Importantly, 8AA treated cells lacked activation of p53 again indicating that the drug worked p53-independently (Figure 7B, lanes 3, 6, and 9).

Our observation of compromised transcription in MANCA and A875 cells after actinomycin D treatment led us to ask if this was due to compromised elongation associated with lower levels of H3K36 trimethylation at transcription start sites (as seen with etoposide treatment in Figure 4). As expected, after actinomycin D treatment we only observed increases in H3K36me3 in ML-1 cells and not in MANCA and A875 cells (Figure 7C). These data support our the data in Figure 4 showing that MANCA and A875 cells had compromised elongation associated with lower H3K36me3 at p53 target genes transcription start sites. However there were large variables in the increase of H3K36me3 in ML-1 cells after treatment, with results from two independent experiments indicating that H3K36me3 increased from approximately 3 to 300 fold for the *p21* gene (Figure 7C represented by error bars). This also occurred for the *puma* gene displaying an approximate 1.5 to 100 fold increase over the control (Figure 7C represented by error bars).

When we compared the outcomes of cells treated with actinomycin D to 8AA, we again documented that the 8AA-induced p53-independent cell death pathway was the most cytotoxic in both T/T and G/G cells. Using an MTT assay, we saw that 8AA caused major significant reductions in mitochondrial activity in ML-1, MANCA and A875 cells (Figure 7D). While actinomycin D treatment was not as effective at reducing mitochondrial activity (Figure 7D). These results clearly indicated that for G/G or T/T cells 8AA was the more effective death activator. In order to observe if 8AA was killing cells through an apoptotic pathway, we performed an Annexin V apoptotic assay followed by flow cytometry. Annexin V is an early marker for apoptosis [66]. Therefore, we compared an early time point of 16 hours of treatment of ML-1, MANCA and A875 cells with 8AA compared to actinomycin D. In ML-1 cells, we observed that this time point for 8AA was even too late to catch early apoptotic markers and that all cells were completely dead (Figure 7E). This corresponded to the observation that there was no actin staining observed for these very dead cells. Overall, we can conclude that 8AA is the most effective at killing T/T and G/G SNP309 cancer cells. These data indicate we should consider p53-independent cell death pathways as primary treatment options.

## DISCUSSION

In cancers with the G/G SNP309 genotype the overexpression of MDM2 causes MDM2-p53 chromatin complexes that we hypothesized would inhibit wild-type p53 mediated transcription initiation [14]. Herein we explored how MDM2 (in G/G SNP309 MANCA and A875 cells) contributed to the compromised transcriptional regulation at p53 target genes. Using chromatin immunoprecipitation experiments we found that the recruitment of p53 to the chromatin caused a recruitment of total and initiated RNA Pol II (Figures 1 and 3). This indicated that the overexpression of MDM2 from the G/G SNP309 genotype did not inhibit transcription initiation. Increased p53 associated with chromatin is able to activate transcription initiation but not transcription elongation at the *p21* gene during the activation of an S phase checkpoint [34–36]. Interestingly, MDM2 induces replication stress eliciting an early intra-S-phase checkpoint response [67]. In p53-null H1299 lung carcinoma cells with inducible wild-type p53 the expression of excess full-length MDM2 selectively inhibits p53 target genes *pig-3* and *14–3-3*σ [68]. We found that stable knockdown of MDM2 moderately increased select p53 target genes in a cell-type specific manner and no additive activation was observed after DNA damage treatment (Figure 2C and data not shown). Our data combined with the published reports on blocks to transcription elongation of p53 target genes after initiation indicate that a complex relationship occurs between p53 protein levels, transcription initiation, and p53-dependent transcriptional regulation. It also suggests that MDM2 may influence transcriptional elongation.

MDM2 inhibits p53-mediated transcription via dual mechanisms by binding to the p53 transactivation domain and also binding to the basal transcription factor TFIIE thereby inhibiting the pre-initiation complex [13, 32]. Our data conflicts with this understanding which was established using an *in vitro* system devoid of chromatin [32]. We observed that G/G SNP309 MDM2 overexpressing cells had functional transcription initiation when treated with the DNA damaging agent etoposide (Figure 3). Therefore, we evaluated markers of transcription elongation in the G/G SNP309 cancer cells to determine if transcription elongation was inhibited (Figure 4). Decreased H3K36me3 associates with decreased transcription elongation [58]. Interestingly, G/G SNP309 A875 and MANCA etoposide and actinomycin D treated cells (when compared to ML-1 T/T treated cells) displayed dramatically lower H3K36me3 for both the *p21* and *puma* genes near transcription start sites (Figures 4C and 7C). Interestingly, two other markers of transcription elongation (RNA Pol II serine-2 and CDK9 recruitment) were not inhibited (Figure 4). While H3K36 reduction associates with decreased transcription elongation it also associates with altered alternative splicing that can associate with a reduced rate of transcription elongation [59, 60]. Our results suggest that chromatin configuration and chromatin modifications impact p53-dependent gene expression but the mechanisms of this inhibition and the association with MDM2 overexpression requires further study.

Regulation of promoter pausing and elongation are key regulatory events during transcription in developmental biology [69]. Human embryonic stem cells have approximately 75% of all genes poised for transcription initiation with regulation at post-initiation steps and only a fraction of the genes able to efficiently elongate [69]. Evidence from Drosophila and some mammalian cell lines show environmental responsive genes are poised for transcriptional activation with paused RNA Pol II near transcription start sites [70, 71]. Deregulation of any number of elongation factors, chromatin remodeling enzymes, histone modifying enzymes and RNA Pol II involved in transcription elongation checkpoint control are linked to multiple diseases including cancer [72]. We asked if increases in H3K36me3 correlated to moderate increases in transcription of p53 target genes in MANCA cells with MDM2 knockdown, which would suggest more active transcriptional elongation. We observed that MDM2 knockdown did not significantly change H3K36me3 at *p21* and *puma* transcription start sites (Figure 5B). Therefore, our data showed that in G/G SNP309 cancer cells MDM2 knockdown was not sufficient to reactivate p53-dependent transcriptional elongation associated with increased H3K36me3.

Although MDM2 is mainly known as an E3-ubiquitin ligase for p53 degradation [12], we observed in the G/G SNP309 cancer cell lines that the excess MDM2 did not lead to decreased p53 protein levels by degradation (Figure 2 and [14]). Interestingly, only MANCA cells had a small significant increase in p53 protein levels with MDM2 knockdown (Figure 2B). We addressed if this was through the ubiquitin-proteasome pathway by treating MANCA cells with MG132 and showed that MDM2 did not increase p53 proteasome-dependent degradation (Figure 2D and E). This demonstrated that MDM2 was not functioning as an E3-ubiquitin ligase for p53 in G/G SNP309 cancer cells. Therefore, MDM2 might regulate p53 translation by indirect mechanisms. For example, MDM2 functions as an E3-ligase for RPL26 promoting its rapid degradation and thereby causes attenuation of p53 mRNA translation [73]. The RING finger domain of MDM2 also directly binds p53 mRNA and enhances p53 translation [74] and the phosphorylation of serine 395 on MDM2 by ATM is necessary for this p53 mRNA-MDM2 interaction [75]. The data presented herein suggests that MDM2 overexpression in G/G SNP309 cancer cells influences the p53 protein level through one of these alternative regulation paradigms.

MDM2 may influence alternative splicing and is itself alternatively spliced [38, 76]. An increase in *mdm2* splice variants and protein isoforms are a common occurrence in cancer cells with MDM2 overexpression (see reviews [38, 77]). Multiple *mdm2* splice variants are found in G/G SNP309 cancer cell lines [78]. The common *mdm2* splice variants found in MDM2 overexpressing cancers express mRNAs that encode polypeptides missing portions of the p53 binding domain, but retain the RING finger domain [77]. Importantly, the G/G SNP309 MANCA cells express high levels of the *mdm2-C* splice transcript as well as high levels of MDM2-C protein [79]. MDM2-C does not increase wild-type p53 degradation or decrease p53 transcriptional activity but rather has p53-independent transforming ability [79]. Therefore, it is possible that knockdown of MDM2 protein in the G/G SNP309 cells did not significantly increase the p53 levels (Figure 2) because some of the MDM2 influenced was MDM2-C. The fact that MDM2 isoforms display both p53-dependent and p53-independent transforming activities further suggests that therapies for cancers with overexpressed MDM2 should be targeted towards p53-independent cell death pathways.

Chemotherapeutic drugs commonly function through DNA damage pathways which signal to p53 [80, 81], but therapeutics that do not damage the DNA can be useful in cases where p53 activity is blocked. DNA damage signals a p53-dependent cascade to induce cell cycle arrest or cell death [6, 7]. Cancer cells with G/G SNP309 MDM2 overexpression are resistant to DNA damaging drugs through an attenuated p53 stress response and therefore require alternative targeted treatments [29, 40]. Small molecule inhibitors have been developed targeting the MDM2-p53 binding pocket as a way to release and activate p53 (see reviews [11, 40, 82]). Nutlin-3 is one such drug that is reported to have efficacy at activating the p53 pathway in MDM2 overexpressing cells with *mdm2* genomic amplification [40, 50]. Activation of the p53 pathway via nucleolar stress as well as DNA damage-independent cancer therapeutics that do not require a p53 activation paradigm can work by inhibiting levels of transcription [62, 83]. Actinomycin D inhibits both Pol I and Pol II transcription [83]. Low dose actinomycin D causes specific activation of the p53 pathway via nucleolar stress [51]. The nucleolus is considered a bull's-eye for cancer therapy because when rRNA synthesis slows drastically p53 levels rise [84]. We used ML-1, MANCA and A875 cells to compare two different modes of interfering with RNA metabolism through treatment with actinomycin D or 8AA. Our results with actinomycin D were in keeping with the published reports that actinomycin D, at low doses, stabilizes p53 in response to ribosomal stress by releasing ribosomal proteins that bind MDM2 [51, 64], but our data indicated that this method was not the most effective in eradicating cancer cells that express wild-type p53. Treatment with 5 nM actinomycin D increased p53 protein levels but had a varied influence on cell viability (Figures 6 and 7). Actinomycin D treatment of ML-1, A875 and MANCA cells increased p53 protein levels but did not robustly inhibit cell viability. Discovering therapeutics that function p53-independently is important because such agents will be useful for cancers regardless of the mechanism by which the p53-pathway is inhibited. The nucleoside analogue 8AA has the promise to be a therapeutic that is excellent for multiple dysfunctional-p53 cancer types [41, 65, 85, 86]. 8AA is able to induce p53-independent cell death in metastatic breast cancers [41]. Importantly, when we treated ML-1, MANCA and A875 cells with 8AA, we observed no changes in p53 protein levels. This indicated that the DNA damage and stress pathways needed to activate p53 were not turned on. More significantly the treatment with 8AA decreased the cell viability of all cell lines tested (Figures 6 and 7). Therefore, inhibiting RNA metabolism using 8AA may be an excellent treatment option for G/G SNP309 MDM2 overexpressing cancers. Clinical trials are already being carried out for 8-chloro-adenosine [62, 87] which works via a similar mechanism. Our study suggests that 8AA should be added to the arsenal of drugs for cancers with high expression of MDM2 and compromised p53 pathways.

## MATERIALS AND METHODS

### Cell culture

ML-1 cells (a generous gift from Michael Kastan), MANCA cells (a generous gift from Andrew Koff) and A875 cells (a generous gift from the Arnold Levine) were grown in RPMI 1640 (Mediatech) with 10% fetal bovine serum (FBS, Gemini) and 50 U/ml penicillin and 50 μg/ml streptomycin (Mediatech) in a 5% CO_2_ 37°C humidified incubator. MANCA and A875 cells were infected with vector containing *mdm2* 151656 shRNA or empty vector by the retroviral gene transfer method. Phoenix packaging cells were transfected with vector containing *mdm2* shRNA or empty vector using the calcium phosphate method in order to make virus used to infect MANCA and A875 cells, then selected with puromycin. The resulting pools were grown under constant selection pressure with 2 μg/mL puromycin for MANCA and 3 μg/mL puromycin for A875 cells. The *mdm2* shRNA oligonucleotide sequences were described in [37]. The MLP vector was a generous gift from Scott Lowe.

### Drug treatments

Cells were treated with: 8 μM etoposide (Sigma-Aldrich) for 6 hours or sterile DMSO (Sigma-Aldrich), 5 nM Actinomycin D (Sigma-Aldrich) for 24 hours and 15 μM 8-amino-adenosine (a generous gift from Dr. Steve Rosen) for 24 hours.

### RNA isolation and quantitative RT-PCR

Cells were centrifuged at 1100 rpm for 7 min at 4°C and washed three times with 1X cold phosphate buffered saline (PBS). The pellets were frozen at −80°C. The RNA was isolated using the RNeasy Mini Kit (Qiagen) according to the manufacturer's protocol and the RNA was stored at −80°C. 1−5 μg of RNA was used to make cDNA with the High Capacity cDNA Archive Kit reagents and protocol (Applied Biosystems). The resulting mix containing RNA, RT buffer, dNTPs, random primers and MultiScribe reverse transcriptase was incubated at 25°C for 10 min and 37°C for 2 hours. Gene transcripts were amplified by quantitative RT-PCR using Taqman Universal Master Mix (Applied Biosystems) and primer probes for *p21* (Hs00355782_m1), *puma* (Hs00248075_m1), *fas* (Hs00538709_m1), *gadd45* (Hs00169255_m1), *pig3* (Hs00153280_m1), and *gapdh* (4333764) or (Hs02758991_g1) (Applied Biosystems on Demand). Quantitative RT-PCR was done with 50–150ng of cDNA from prep. The 7500 Sequence Detection System or ViiA 7 Real Time PCR system (Applied Biosystems) followed the program: one cycle, 2 min, 50°C; one cycle, 10 min, 95°C; and 40 cycles, 15 seconds, 95°C and 1 minute 60°C.

### Whole cell extracts

Cells were pelleted at 1100 rpm for 7 min at 4°C and washed three times with 1X ice-cold PBS. The cell pellets were resuspended in RIPA buffer (0.1% SDS, 1% IGEPAL NP-40, 0.5% Deoxycholate, 150 mM NaCl, 1 mM EDTA, 0.5 mM EGTA, 50 mM Tris-Cl pH 8.0, 1 mM PMSF, 8.5 μg/ml Aprotinin (Sigma-Aldrich), and 2 μg/ml Leupeptin)was incubated on ice for 60 minutes to lyse the cells with vortexing every 5–10 min. Additional sonication of lysate 3 times for 30 seconds/30 seconds rest on ice was done. Samples centrifuged at 13,000 rpm for 20 min at 4°C. The supernatants were stored at −80°C.

### Chromatin fractionation

Cells were pelleted at 1100 rpm for 7 min at 4°C and washed three times with 1X ice-cold PBS. Cells were suspended in Buffer A (10 mM HEPES pH 7.9, 10 mM KCl, 1.5 mM MgCl2, 0.34M Sucrose, 10% glycerol, 1 mM DTT, 0.5 mM PMSF, 2 μg/ml Leupeptin, 8.5 μg/ml Aprotinin) + 0.1% Triton X-100. Incubated on ice 5 min. Spun down cells 3600 rpm for 5 min at 4°C. Spun down supernatant for an additional 5min at 13,000 rpm at 4°C to clarify (S1 Fraction). Washed pellet 2 times with Buffer A spinning down at 3600 rpm for 5min at 4°C. Resuspended nuclei pellet in Buffer B (3 mM EDTA, 0.2 mM EGTA, 0.5 mM PMSF, 2 μg/ml Leupeptin, 8.5 μg/ml Aprotinin). Samples were incubated on ice 30 minutes with vigorous vortexing every 5 minutes and then spun down 4000 rpm for 5 min at 4°C. The supernatant is nuclear soluble proteins (S2 Fraction) and the pellet is enriched in chromatin. Washed pellet 2 times with Buffer B. Resuspended pellet (P3 Fraction) in RIPA buffer and sonicated 3 times 30 sec/30 sec rest on ice. Froze samples at −80°C.

### Western blot analysis

Cell protein samples were prepared with 4X NuPAGE Lithium Dodecyl Sulfate buffer (Life Technologies) and 20 mM DTT. The samples were heated at 70°C for 10 min and then 100 mM Iodoacetamide (Sigma-Aldrich) was added after heating. Samples were separated by SDS-PAGE followed by electrotransfer onto a nitrocellulose membrane. The resulting membrane was blocked using 5% non-fat milk (BioRad) in 1X PBS-0.1% Tween-20 and probed with primary antibody overnight at 4°C. The membrane was washed with 1X PBS-0.1% Tween-20 solution followed by probing with secondary antibody. The signal was detected by chemiluminescence with the Super Signal Kit (Pierce) and autoradiography using Hyblot CL films (Denville Scientific).

### Chromatin immunoprecipitation

Cells were cross-linked for 10 min at 37°C with 1% formaldehyde followed by addition of 0.125 M Glycine to quench the reaction with 5 min of shaking at room temperature. Cells were washed four times with cold 1X PBS and centrifuged at 1100 rpm for 7 min at 4°C for suspension cells and 2000 rpm for 15 min at 4°C for adherent cells. The cell pellets were resuspended in 1X RIPA Buffer (with 1/100 dilution phosphatase inhibitor cocktail 3 (Sigma-Aldrich)) and incubated for 30 minutes on ice. The samples were sonicated (1min/1min rest at 98% amplitude) on ice to shear DNA into approximately 3500–150 bp fragments followed by centrifugation at 13,000 rpm for 20 min at 4°C. Immunoprecipitations were carried out overnight using 400 μg of protein. Protein A/G Plus Agarose beads (Santa Cruz) (washed in PBS and blocked with 0.3 mg/ml herring sperm DNA (Invitrogen) for 30 min at 4°C) were added for 2 hrs with rocking at 4°C. Anti-mouse IgM Agarose beads (Sigma) used for IgM based antibodies (phospho-Serine 5 CTD RNA pol II (Covance) and mouse IgM Isotype control(SIGMA)) and diluted to 25% bead slurry and blocked with 1mg/ml BSA and 0.3mg/ml herring sperm DNA. The samples were centrifuged for 2 min at 3,000 rpm at 4°C and washed with (1) 0.1% SDS, 1% Triton-X, 20 mM Tris pH 8.1, 150 mM NaCl, (2) same as Wash 1 but NaCl is 500 mM, (3) 0.25 M LiCl, 1%IGEPAL, 1% Na Deoxycholate, 1 mM EDTA, 10 mM Tris pH 8, (4) TE pH 8, (5) same as Wash 4; spinning the beads down between each wash. Elution buffer (0.1M NaHCO_3_ in 1% SDS) and 1mg/ml Proteinase K (Sigma-Aldrich) was added to beads and incubated overnight at 65°C. 40 μg of cell lysate was used as total DNA input. The DNA fragments were purified using the PCR Purification kit (Qiagen) according to manufacturer's protocol and amplified by quantitative PCR.

Primer probe mixes used with Taqman Universal Master Mix as described in [88, 89]:

*p21* p53RE (5′):

Forward- GTGGCTCTGATTGGCTTTCTG

Reverse- CTGAAAACAGGCAGCCCAAG

Probe- TGGCATAGAAGAGGCTGGTGGC TATTTTG

*p21* TATA box:

Forward- CGCGAGGATGCGTGTTC

Reverse CATTCACCTGCCGCAGAAA

Probe - CGGGTGTGTGC

*puma* p53RE:

Forward-GCGAGACTGTGGCCTTGTGT

Reverse- CGTTCCAGGGTCCACAAAGT

Probe- TGTGAGTACATCCTCTGGGCTC TGCCTG

Primers used with SYBR Green PCR Master Mix (Applied Biosystems) as described in [90, 91]:

*p21*+7011 F- CCTGGCTGACTTCTGCTGTCT

*p21*+7011 R- CGGCGTTTGGAGTGGTAGA

*puma* +6014 F-AGGTGCTGCTCCGCCA

*puma* +6014 R- CCCTCTGCCTCTCCAAGGTC

### Antibodies

For Western blot analysis: MDM2 – 1:1:1 mix of mouse monoclonal antibodies 4B2,2A9,4B11; p53–1:1:1 mix of mouse monoclonal antibodies 240,421,1801;, goat polyclonal H3 (C-16) (Santa Cruz) rabbit polyclonal anti-Actin (Sigma-Aldrich) or anti-Actin HRP (Santa Cruz); secondary anti-mouse, anti-goat/sheep and anti-rabbit antibodies (Sigma-Aldrich)

For ChIP: (DO-1) mouse monoclonal anti-p53 (Calbiochem), (N-20) rabbit polyclonal anti-Mdm2 (Santa Cruz); (H-224) rabbit polyclonal Pol II (Santa Cruz); (H5) mouse monoclonal anti-phospho Serine 5 CTD RNA pol II (Covance); rabbit polyclonal anti-RNA polymerase II CTD repeat (phospho S2) ChIP grade (ab5095-abcam); (C-20) rabbit polyclonal Cdk9 (Santa Cruz); rabbit polyclonal anti-Histone H3K36me3 ChIP grade (ab9050-abcam); Rabbit IgG (Santa Cruz); Mouse IgG (Santa Cruz); Mouse IgM isotype control (Sigma-Aldrich)

### MTT assay

MTT (3-(4,5-Dimethylthiazol-2-yl)-2,5-diphenyltetrazolium bromide) stock solution (5mg/ml in clear HANKs media) was added to treated cells in volume equal to 10% of culture medium followed by incubation at 37°C until color developed. Then an equal volume of solubilization solution was added (90% anhydrous isopropanol, 10% Triton X-100, and. 826% 12.1 N HCl). Samples were analyzed in the plate reader. The absorbance was measured at 550nm and the 620nm absorbance was subtracted for background to allow for quantification.

### Annexin V apoptosis assay

ApoScreen Annexin V Apoptosis assay (Southern Biotech Cat. No. 10010–02) was used according to manufacturer's protocol. After 16 hours of drug treatment, ML-1 and MANCA cells spun down at 1100 rpm for 7 min at 4°C and A875 cells were trypsinized off plates followed by centrifugation. The cells were washed twice with 1X cold PBS and resuspended in binding buffer provided by manufacturer. Cells were incubated with 10 μL of Annexin V-FITC reagent for 15 min on ice. This was followed by 10 μL of propidium iodide and then flow cytometry analysis. Flourescence-activated cell sorting (FACS) was performed on a BD Bioscience FACS scan.
